# Preoperative Neutrophil-to-Lymphocyte Ratio and Neutrophilia Are Independent Predictors of Recurrence in Patients with Localized Papillary Renal Cell Carcinoma

**DOI:** 10.1155/2015/891045

**Published:** 2015-09-03

**Authors:** Jiwei Huang, Douglas M. Dahl, Liang Dong, Qiang Liu, Kristine Cornejo, Qi Wang, Shulin Wu, Adam S. Feldman, Yiran Huang, Wei Xue, Chin-Lee Wu

**Affiliations:** ^1^Department of Urology, Renji Hospital, School of Medicine, Shanghai Jiao Tong University, Shanghai 200123, China; ^2^Department of Urology, Massachusetts General Hospital and Harvard Medical School, Boston, MA 02114, USA; ^3^Department of Pathology, Renji Hospital, School of Medicine, Shanghai Jiao Tong University, Shanghai 200123, China; ^4^Department of Pathology, Massachusetts General Hospital and Harvard Medical School, Boston, MA 02114, USA; ^5^Department of Pathology, Boston Children's Hospital and Harvard Medical School, Boston, MA 02115, USA

## Abstract

*Objective*. To evaluate the role of preoperative neutrophil-to-lymphocyte ratio (NLR) and absolute neutrophil count (ANC) in patients' prognosis with localized papillary renal cell carcinoma (pRCC). *Methods*. Data from 218 localized pRCC patients (T1-3 N0/+ M0), operated between 1991 and 2011 at two centers, were evaluated retrospectively. Univariable and multivariable analyses using the Cox regression model were performed to determine the associations of NLR and ANC with recurrence-free survival (RFS). Prognostic accuracy was evaluated with the Harrell concordance index. *Results*. The 5-year RFS rate was 87.0%. Multivariable analysis identified increased NLR (≥3.6) and ANC (≥5300/*μ*L) as independent prognostic factors for RFS (hazard ratio (HR) = 4.01, *P* = 0.018) and (HR = 4.71, *P* = 0.045). The final model built by the addition of NLR or ANC improved predictive accuracy (c-index: 0.824, 0.842) compared with the clinicopathological base model (c-index: 0.800), which included TNM stage and tumor necrosis. *Conclusions*. The NLR and ANC appear to be independent prognostic factors for RFS after surgery for localized pRCC. They significantly increase the accuracy of established prognostic factors. Therefore, we recommend adding NLR and ANC to traditional prognostic model, which may improve its predictive accuracy.

## 1. Introduction

Papillary renal cell carcinoma (pRCC) accounts for approximately 10% to 15% of all renal cell carcinoma (RCC) and is the second most frequent histologic form after clear cell RCC (ccRCC) [1, 2]. Because clear cell histology accounts for approximately 70–80% of all RCCs, most data regarding prognostic factors have been served for this subtype. It is important to note that a particular histologic subtype is characterized by distinct morphological features, clinical behaviors, and genetic changes [1–3]. Patients with pRCC have a significantly better prognosis than those with clear cell RCC but some patients with pRCC die of metastatic disease [1, 2]. Because of the relatively lower frequency of recurrence, it is also complicated to identify prognostic factors in pRCC compared with ccRCC. It is accurately crucial to determine the risk of disease recurrence in the postoperative phase since it impacts the frequency and extent of surveillance imaging and possible inclusion in adjuvant clinical trial [4]. Therefore, particular prognostic factors and models should be independently validated for pRCC.

Several prognostic factors and models were proposed to determine the risk of disease recurrence of RCC [5–7]. The systemic inflammatory response, which is usually measured by surrogate blood-based parameters, such as neutrophil or platelet count, C-reactive protein, has been shown to independently predict the clinical outcome of various human cancer types [8]. Neutrophil-to-lymphocyte ratio (NLR) could serve as independent predictors of survival [9–12] in ccRCC patients or in RCC patients, which were composed of overwhelming majority of ccRCCs. Only one study investigated NLR in nonclear cell RCC, but they did not analyze it in separate histologic subtype [13]. One recent study focused on ALC in pRCC [14].

To our knowledge, the prognostic value of absolute neutrophil count (ANC), NLR in pRCC has not been investigated. Compared to ccRCC, pRCC involves different biological pathways, has distinct prognostic factors, and is associated with a more favorable prognosis [2]. We therefore evaluated the prognostic value of preoperative ANC, absolute lymphocyte count (ALC), and NLR, which are routinely measured, in localized pRCC patients treated with curative intent surgery.

## 2. Materials and Methods

### 2.1. Study Population

**Figure 1:**
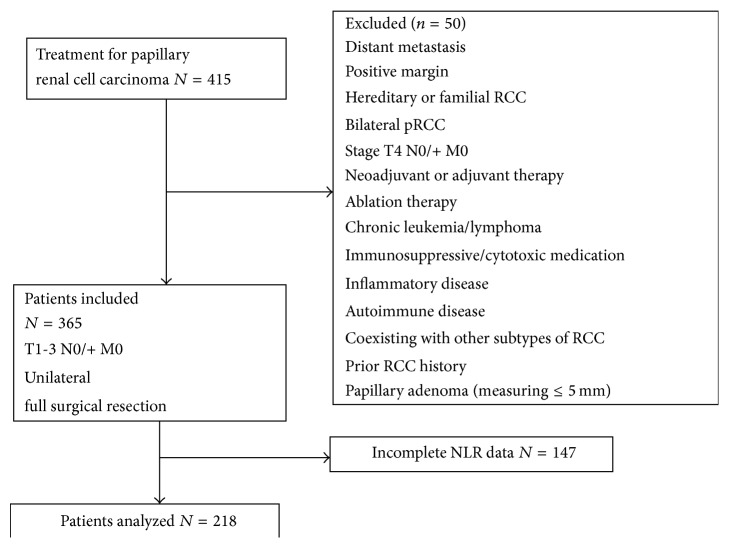
Flow chart of patients who met study inclusion/exclusion criteria.

After obtaining the institutional review board (IRB) approval from both hospitals, we retrospectively reviewed 415 consecutive patients with pRCC who underwent radical or partial nephrectomy for pRCC between 1991 and 2011 at two academic centers. We finally included 218 patients who underwent full resection of unilateral, sporadic pRCC (stage T1-3N0M0 or T1-3N+M0) by radical or partial nephrectomy with available data on ALC and ANC within 4 weeks before surgery. We excluded patients with tumor measuring 5 mm or less (papillary adenoma), those with chronic leukemia or lymphoma, inflammatory disease, and autoimmune disease, those receiving neoadjuvant or adjuvant therapy, those with coexisting other subtypes of RCC, and those with a prior history of RCC.  Figure 1 shows a flow chart of patients who met inclusion criteria.

### 2.2. Clinical and Pathological Evaluation

Patients' data including age, gender, symptoms at presentation, hypertension, diabetes mellitus, pathological features, ANC, ALC, and follow-up information were collected. All clinical data were retrieved from medical records at both institutions. The pathologic slides were rereviewed by the urologic pathologists at the individual institution (Kristine Cornejo and Chin-Lee Wu from Massachusetts General Hospital, and Qiang Liu from Renji Hospital). In total, 6 pathological slides could not be found in two institutions. Their pathological data were retrieved from pathology records at two hospitals. Patients with a palpable flank or abdominal mass, discomfort, gross hematuria, acute onset varicocele, or constitutional symptoms, including sweating, weight loss, fatigue, early satiety, rash, and anorexia, were considered symptomatic at presentation. For preoperative staging patients underwent computed tomography (CT) or magnetic resonance imaging (MRI) of the abdomen and CT or X-ray of the chest. Radionuclide bone scan and cranial CT were performed as clinically indicated. TNM staging was performed according to 2010 criteria [15], T, N stages were assigned pathologically and M stage was assigned clinically. Patients with pathological nodes on imaging underwent lymph node dissection. Pathological confirmation was done in all study patients with positive nodes. Histological differentiation was graded according to Fuhrman's nuclear grading system [16]. Each lesion was also classified as type 1 or 2 based on the features used to describe pRCC in the original study by Delahunt and Eble [17]. Any tumor with type 2 areas was classified as type 2.

### 2.3. Follow-Up

The postoperative surveillance strategy was institution and physician dependent. Recurrence was defined as local relapse, lymph-node metastasis, and distant metastasis, as determined by imaging (mainly CT, MRI, and bone scan) and most of which was confirmed by pathology.

### 2.4. Laboratory Assays

Venous blood samples were collected as part of routine clinical procedures before surgery at both institutions and laboratory parameters for this study were obtained from medical records. The cutoff points to stratify ANC, ALC, and NLR were using ANC < 5,300 cells per *μ*L to identify low ANC, ALC < 1,300 cells per *μ*L to identify low ALC, and NLR < 3.6 to identify low NLR [13].

### 2.5. Statistical Analysis

As to the clinicopathological outcomes according to the ANC, ALC, and NLR, for continuous variables, Student's *t*-test was used for those variables reported as mean (plus or minus standard deviation [SD]) and Wilcoxon rank-sum test was used for the variables reported as median with interquartile ranges (IQRs); for categorical variables, the chi-square and continuity corrected chi-square tests were used. The primary outcome of this study, recurrence-free survival (RFS), was estimated by the Kaplan-Meier method and compared by a log-rank test and calculated from the date of surgery to the date of disease recurrence or the time of the last visit. The effect of ANC, ALC, and NLR on RFS was examined by using a Cox proportional hazard regression model. All variables including ANC, NLR with a *P* less than 0.05 on univariable analyses were entered into multivariable stepwise Cox regression analysis. To prevent against overfitting by decreasing the number of variables, pT stage and pN stage were summarized as the TNM group in multivariable analysis. Hazard ratio (HR) and 95% confidence interval (CI) were computed. The predictive accuracy was evaluated using the Harrell concordance index (c-index) and given as a percentage [18]. A clinicopathological base model was built, consisting of all variables that demonstrated a significant independent prognostic value in the multivariable Cox proportional hazard regression model. To examine whether ANC or NLR data can provide additional prognostic power when used with basic clinical variables, we built predictive models by integrating clinical variables with ANC or/and NLR data using the statistical method described in [19]. For each core set, we randomly split the samples into two groups: 80% as the training set and 20% as the test set. The multivariate Cox models were built based on training set with the R package “survival.” We then applied the models thereby obtained to the test set for prediction and calculated the c-index from test set using the R package “survival.” For each core set, the above procedure was repeated 100 times to generate 100 c-indexes. Then, we used the Wilcoxon signed rank test to calculate the *P* value (using 0.05 as the significance cutoff). The differences were considered to be statistically significant if *P* < 0.05. Statistical analysis was carried out using SPSS, version 22.0.

## 3. Results

### 3.1. Clinical Characteristics

Our final cohort included 169 men (77.5%) and 49 women (22.5%). Mean age at surgery was 58.9 years (Table 1). Radical and partial nephrectomy was performed in 129 (59.2%) and 89 patients (40.8%), respectively.

### 3.2. Associations

#### 3.2.1. With Clinical and Pathological Characteristics

The median preoperative ANC, ALC, and NLR was 5.3/nL (IQR: 4.2, 7.0), 1.7/nL (IQR: 1.4, 2.1), and 3.1 (IQR: 2.4, 4.2). Compared with patients with lower ANC (<5.3/nL) and NLR (<3.6), patients with higher ANC and NLR were more likely to have larger tumor size (*P* = 0.044, *P* = 0.005) (Tables 1–3). There were no significant differences between the groups according to ANC, ALC, and NLR with regard to other established prognostic factors, such as pathological stage, symptoms at diagnosis. Of host related factors, neutrophilia was only associated with gender (*P* = 0.038).

#### 3.2.2. With Prognosis of pRCC

At a median follow-up period of 43.0 months (IQR 17.8–67.5, mean 52.8), disease recurrence occurred in 20 patients; the 5-year disease-free survival rate was 87.0%.

**Figure 2:**
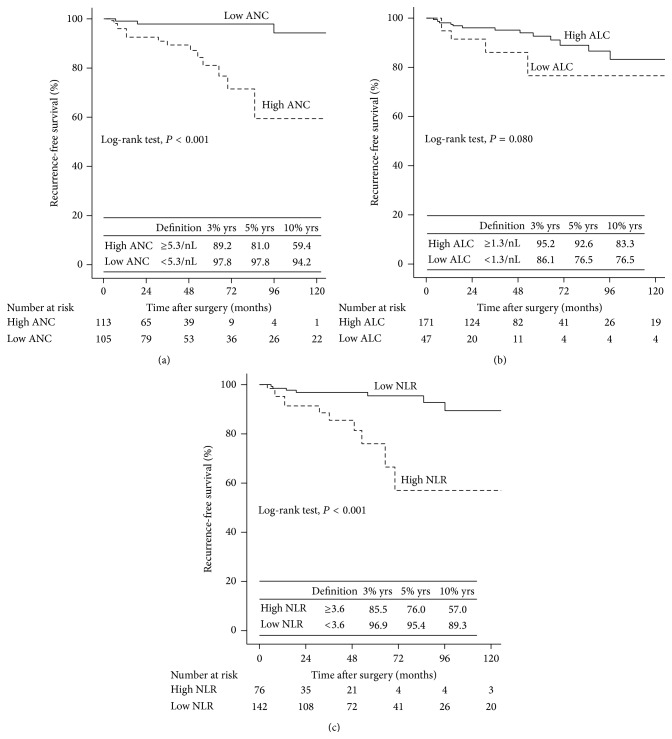
Kaplan-Meier curves for pRCC patients RFS groups categorized (a) by ANC, (b) ALC, and (c) NLR.

The patients with preoperative neutrophilia and high NLR had a significantly worse rate of survival than those without neutrophilia and high NLR with regard to RFS (Log-rank test, with each *P* < 0.001, Figure 2). Univariable and multivariable analyses (stepwise analysis) of the factors influencing RFS are presented in Table 4. Univariable analyses demonstrated that pT stage, pN stage, TNM stage, Fuhrman grade, pRCC type, tumor necrosis, neutrophilia, and high NLR were significant predictors of RFS. Multivariable analyses showed that neutrophilia (HR 4.71, *P* = 0.045) and high NLR (HR 4.01, *P* = 0.018) were independent predictors of RFS, along with the presence of TNM stage (HR 2.19, *P* = 0.003) and tumor necrosis (HR 2.55, *P* = 0.057).

**Figure 3:**
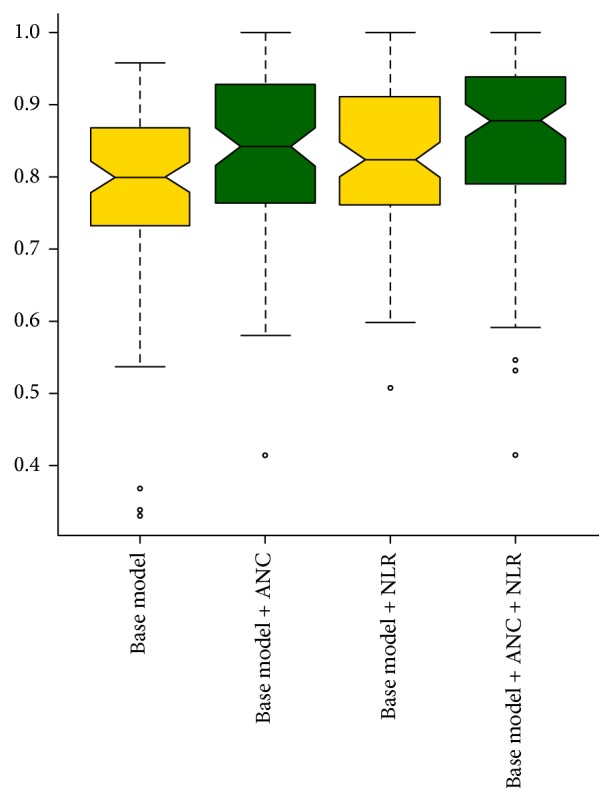
Comparison of the survival predictive power of base model (TNM stage + tumor necrosis) and integrated model combined with ANC, NLR.

The predictive accuracy was calculated with and without the inclusion of ANC and NLR. In the base model, including the traditional predictor variables of TNM stage and tumor necrosis, predictive accuracy was 80.0%; with the addition of ANC 5.3/nL, predictive accuracy was 84.2%; with the addition of NLR 3.6, predictive accuracy was 82.4%. In a model including all four variables, predictive accuracy was 87.8% (Table 5). Notably, the integrated models resulted in statistically significantly improved predictive power compared to the base model (one-sided Wilcoxon signed rank test, TNM stage + tumor necrosis + ANC: *P* < 7.5 × 10^−4^; TNM stage + tumor necrosis + NLR: *P* < 2.5 × 10^−3^; TNM stage + tumor necrosis + NLR: *P* < 5.3 × 10^−6^) (Figure 3).

## 4. Discussion

This study showed that ANC and NLR were independent prognostic factors after surgery with curative intent for localized pRCC and found that both of them, especially NLR, could significantly increase the accuracy of established prognostic model. Therefore, NLR and ANC may improve the predictive accuracy of traditional prognostic model.

To our knowledge, this is the first study of NLR focusing on pRCC. In published studies to date, only patients with ccRCC were included or the subtypes were predominantly clear cell. Only one study investigated NLR in nonclear cell RCC and did not access it in papillary cell subtype. In a cohort of 678 patients with nonmetastatic ccRCC, Pichler et al. [10] found that preoperative NLR was an independent prognostic factor for overall survival (HR 1.59; *P* = 0.014). de Martino et al. [13] showed that patients with increased preoperative NLR, evaluated as a continuous variable, had a high risk of disease recurrence in nonclear cell RCC. Mehrazin et al. [14] demonstrated that patients with lymphopenia had an inferior overall survival and a trend significance for worse cancer-specific survival (*P* = 0.071) in pRCC.

In the present study, patients who had both pRCC and high NLR or neutrophilia were more likely to have larger tumor size. de Martino et al. reported that patients with increasing ANC and NLR were associated with lymph node metastasis. We found a nonsignificant trend in favor of increasing NLR associated with lymph node metastasis (*P* = 0.097). These differences of tumor characteristics may partly explain why the patients with high NLR or neutrophilia in our cohort had more aggressive disease.

Despite the fact that recent progress in the identification of genetic and common molecular alterations in RCC has been made [20], the most widely used routine prognostic assessment of RCC currently still relies on traditional clinicopathological prognostic variables [5–7]. The predictive accuracy of prognostic model can be improved by these molecular markers, but the high costs of analysis, the time-consuming preparation, and the lack of evidence together turn them into clinical practice. At the meantime, several inflammatory hematological indexes, such as C-reactive protein [21], have been proved to improve the accuracy of these models in patients with RCC. In our study, we also showed that adding NLR or ANC was able to raise the predictive accuracy in this cohort of patients. The base model, which included the traditional predictor variables of TNM stage and tumor necrosis, was of a predictive accuracy (80.0%), which could be further improved by the addition of NLR (82.4%) or ANC (84.2%). Our findings are in agreement with the study of de Martino et al. [13], who also found that adding NLR to their base model (TNM stage, grade and microvascular invasion) could improve the predictive accuracy from 78.8% to 80.8% in 281 nonclear cell RCC patients with regard to RFS. Considering NLR that is widely available and relatively easy to assess even before surgery, they may become attractive variables for patients counseling and clinical trial entry.

Increasing evidence supports the involvement of systemic inflammation in cancer development and progression. On one hand, inflammation and activation of the immune system enable antitumor activity; on the other hand, they contribute to carcinogenesis, tumor growth, and progression in human cancers [22]. Local inflammation, which is reflected by intratumoral infiltration of neutrophils, macrophages, and leucocytes, was also identified as an independent factor for reduced survival in clear cell RCC patients [23]. In our opinion, inflammation no matter systemic or local contributing to carcinogenesis may be the underlying reason why NLR, neutrophil count, and local intratumoral neutrophil presence are significantly associated with the poor outcome of the RCC patients.

The present study has several limitations, including the retrospective nature of the data collection and data limited to 2 centers. There was no standard for postoperative surveillance, which may have impact on the outcome measurement and subsequent statistical evaluation. Although pathology was rereviewed by urologic pathologists at individual institution, we did not provide a central pathology review. Also, some other prognostic factors, such as performance status score were not evaluated in this study. In addition to the evaluation of the integration of the NLR and ANC to our base model, other prognostic models such as the Kattan nomogram [5] or the UISS model [6] should be evaluated. Nonetheless, even considering these limitations, our data clearly indicate that an increased pretreatment of NLR and ANC might represent an independent prognostic factor for RFS in localized pRCC patients.

## 5. Conclusions

In conclusion, an increased NLR and ANC are independent prognostic factors for RFS after surgery with curative intent for localized pRCC. They significantly increase the predictive accuracy of established prognostic factors. Therefore, we recommend adding NLR and ANC to traditional prognostic model, which may improve its predictive accuracy.

## 6. Clinical Practice Points

Papillary renal cell carcinoma (pRCC) is the second most frequent histologic form of RCC and is characterized by distinct clinical behaviors and better outcome compared with ccRCC. Although neutrophil-to-lymphocyte ratio (NLR) was showed to serve as independent predictor of survival in ccRCC or RCC patients, which were composed of overwhelming majority of patients, the prognostic value of absolute neutrophil count (ANC), NLR in pRCC has not been investigated.

The findings of this study indicate that compared with patients with lower NLR and ANC, patients with higher NLR and ANC were more likely to have larger tumor size. NLR and ANC were independent prognostic factors for RFS of localized pRCC. Adding each factor into the clinicopathological base model could improve the predictive accuracy.

We recommend adding NLR and ANC to traditional prognostic model, which may improve its predictive accuracy. However, until now, NLR and ANC have not been yet included in well-known prognostic models for localized ccRCC, such as UISS, SSIGN models. So, there is still a long way to go to include preoperative NLR and ANC in prognostic models for pRCC patients.

## Figures and Tables

**Table 1:** Clinical and pathological characteristics of 218 pRCC patients stratified according to NLR.

Variables	Number of Pts (%)	NLR ≥ 3.6	NLR < 3.6	*P* value
Patients, *n* (%)	218	76 (34.9)	142 (65.1)	
Age (years), mean ± SD	58.9 ± 12.2	59.7 ± 11.6	58.5 ± 12.5	0.460
Gender				0.712
Male	169 (77.5)	60 (78.9)	109 (76.8)
Female	49 (22.5)	16 (21.1)	33 (23.2)
Symptoms at presentation				0.140
Symptom	37 (17.0)	9 (11.8)	28 (19.7)
Asymptomatic	181 (83.0)	67 (88.2)	114 (80.3)
Hypertension				0.053
Yes	91 (41.7)	25 (32.9)	66 (46.5)
No	127 (58.3)	51 (67.1)	76 (53.5)
Diabetes mellitus				0.431
Yes	22 (10.1)	6 (7.9)	16 (11.3)
No	196 (89.9)	70 (92.1)	126 (88.7)
Tumor size (cm), median (IQR)	3.5 (2.5–6.0)	4.0 (3.0–7.0)	3.2 (2.1–5.0)	0.005
T stage				0.290
T1	160 (73.4)	52 (68.4)	108 (76.1)
T2	20 (9.2)	10 (13.2)	10 (7.0)
T3	38 (19.5)	14 (18.4)	24 (16.9)
N stage				0.097
N1	7 (3.2)	5 (6.6)	2 (1.4)
N0	211 (96.8)	71 (93.4)	140 (98.6)
Fuhrman grade				0.763
1-2	155 (71.1)	55 (72.4)	100 (70.4)
3-4	63 (28.9)	21 (27.6)	42 (29.6)
pRCC type				0.079
1 type	126 (57.8)	38 (51.4)	88 (63.8)
2 type	86 (39.4)	36 (48.6)	50 (36.2)
Unknown	6 (2.8)			
Tumor necrosis				0.653
Yes	34 (15.6)	13 (17.1)	21 (14.8)
No	184 (84.4)	63 (82.9)	121 (85.2)

**Table 2 tab2:** Clinical and pathological characteristics of 218 pRCC patients stratified according to ANC.

Variables	ANC ≥ 5.3/nL	ANC < 5.3/nL	*P* value
Patients, *n* (%)	113 (51.8)	105 (48.2)	
Age (years), mean ± SD	59.3 ± 12.2	58.5 ± 12.3	0.614
Gender			0.038
Male	94 (83.2)	75 (71.4)
Female	19 (16.8)	30 (28.6)
Symptoms at presentation			0.131
Symptom	15 (13.3)	22 (21.0)
Asymptomatic	98 (86.7)	83 (79.0)
Hypertension			0.090
Yes	41 (36.3)	50 (47.6)
No	72 (63.7)	55 (52.4)
Diabetes mellitus			0.279
Yes	9 (8.0)	13 (12.4)
No	104 (92.0)	92 (87.6)
Tumor size (cm), median (IQR)	4.0 (2.5–6.6)	3.5 (2.5–4.8)	0.044
T stage			0.130
T1	77 (68.1)	83 (79.0)
T2	14 (12.4)	6 (5.7)
T3	22 (19.5)	16 (15.3)
N stage			0.150
N1	6 (5.3)	1 (1.0)
N0	107 (94.7)	104 (99.0)
Fuhrman grade			0.918
1-2	80 (70.8)	75 (71.4)
3-4	33 (29.2)	30 (28.6)
pRCC type			0.119
1 type	61 (54.5)	65 (65.0)
2 type	51 (45.5)	35 (35.0)
Tumor necrosis			0.888
Yes	18 (15.9)	16 (15.2)
No	95 (84.1)	89 (84.8)

**Table 3:** Clinical and pathological characteristics of 218 pRCC patients stratified according to ALC.

Variables	ALC ≥ 1.3/nL	ANC < 1.3/nL	*P* value
Patients, *n* (%)	171 (78.4)	47 (21.6)	
Age (years), mean ± SD	58.1 ± 11.9	62.0 ± 12.9	0.053
Gender			0.863
Male	133 (77.8)	36 (76.6)
Female	38 (22.2)	11 (23.4)
Symptoms at presentation			0.992
Symptom	29 (17.0)	8 (17.0)
Asymptomatic	142 (83.0)	39 (83.0)
Hypertension			0.227
Yes	75 (43.9)	16 (34.0)
No	96 (56.1)	31 (66.0)
Diabetes mellitus			1.000
Yes	17 (9.9)	5 (10.6)
No	154 (90.1)	42 (89.4)
Tumor size (cm), median (IQR)	3.5 (2.3–6.0)	4.0 (3.0–5.5)	0.050
T stage			0.831
T1	125 (73.1)	35 (74.5)
T2	15 (8.8)	5 (10.6)
T3	31 (18.1)	7 (14.9)
N stage			1.000
N1	5 (2.9)	2 (4.3)
N0	166 (97.1)	45 (95.7)
Fuhrman grade			0.565
1-2	120 (70.2)	35 (74.5)
3-4	51 (29.8)	12 (25.5)
pRCC type			0.551
1 type	101 (60.5)	25 (55.6)
2 type	66 (39.5)	20 (44.4)
Tumor necrosis			0.761
Yes	26 (15.2)	8 (17.0)
No	145 (84.8)	39 (83.0)

**Table 4:** Univariable and multivariable Cox regression models to predict RFS in 218 patients treated with nephrectomy with curative intent for pRCC.

Variables	Univariable	*P* value	Stepwise analyses	*P* value
	HR (95% CI)		HR (95% CI)	
Age greater than 60	0.74 (0.31–1.79)	0.507		
Gender (male versus female)	0.92 (0.34–2.54)	0.875		
Symptoms at presentation (yes versus no)	2.07 (0.79–5.41)	0.138		
Hypertension (yes versus no)	1.40 (0.56–3.46)	0.471		
Diabetes mellitus (yes versus no)	0.43 (0.06–3.24)	0.414		
pTNM stage				
T (III versus II versus I)	2.43 (1.50–3.92)	<0.001		
N1 versus pNx/0	5.82 (1.29–26.28)	0.022		
TNM group (III versus II versus I)	2.62 (1.62–4.26)	<0.001	2.19 (1.31–3.64)	0.003
Grade (G3-4 versus G1-2)	3.24 (1.31–7.99)	0.011	—	—
pRCC type (type 2 versus 1)	3.07 (1.21–7.77)	0.018	—	—
Tumor necrosis (yes versus no)	3.10 (1.23–7.80)	0.016	2.55 (0.97–6.70)	0.057
ANC (≥5.3/nL versus <5.3/nL)	8.64 (2.39–31.15)	0.001	4.71 (1.04–21.35)	0.045
ALC (≥1.3/nL versus <1.3/nL)	0.43 (0.16–1.14)	0.089		
NLR (≥3.6 versus <3.6)	5.27 (2.11–13.17)	<0.001	4.01 (1.26–12.73)	0.018

**Table 5:** Multivariable model of possible independent prognostic variables in pRCC patients.

Covariable	Categories	Multivariable	*P* value	Multivariable	*P* value	Multivariable	*P* value	Multivariable	*P* value
		HR (95% CI)		HR (95% CI)		HR (95% CI)		HR (95% CI)	
TNM stage	III versus II versus I	2.43 (1.49–3.98)	<0.001	2.34 (1.41–3.89)	0.001	2.39 (1.44–3.96)	0.001	2.21 (1.33–3.68)	0.002
Tumor necrosis	Yes versus no	2.19 (0.85–5.64)	0.103	2.72 (1.04–7.14)	0.042	2.68 (1.03–7.01)	0.044	2.83 (1.08–7.39)	0.034
ANC	≥5.3 versus <5.3			9.64 (2.46–37.76)	0.001			6.04 (1.38–26.40)	0.017
NLR	≥3.6 versus <3.6					5.98 (2.24–15.98)	<0.001	3.18 (1.12–9.05)	0.030
Predictive accuracy (%)		80.0		84.2		82.4		87.8	
